# A Fast Identification Method for Seismic Responses of Bridge Structures by Integrating Digital Signal Features and Deep Learning

**DOI:** 10.3390/s25020399

**Published:** 2025-01-11

**Authors:** Zhaoxu Lv, Youliang Ding, Junxiao Guo

**Affiliations:** 1 Jiangsu Xiandai Road & Bridge Co., Ltd., Nanjing 210018, China; 2 Key Laboratory of Concrete and Pre-Stressed Concrete Structures of the Ministry of Education, Southeast University, Nanjing 210096, China; 3 College of Civil Engineering, Southeast University, Nanjing 210096, China

**Keywords:** bridge structures, seismic identification, signal processing, deep learning

## Abstract

A method of bridge structure seismic response identification combining signal processing technology and deep learning technology is proposed. The short-time energy method is used to intelligently extract the non-smooth segments in the sensor acquired signals, and the short-time Fourier transform, continuous wavelet transform, and Meier frequency cestrum coefficients are used to analyze the spectrum of the non-smooth segments of the response of the bridge structure, and the response feature matrix is extracted and used to classify sequences or images in the LSTM network and the Resnet50 network. The results show that the signal processing techniques can effectively extract the structural response features and reduce the overfitting phenomenon of neural networks, and the combination of signal processing techniques and deep learning techniques can recognize the seismic response of bridge structures with high accuracy and efficiency.

## 1. Introduction

With the continuous advancement of infrastructure construction, many bridges have been built and put into operation, significantly improving transportation efficiency. However, these critical structures, while providing significant benefits, are also susceptible to extreme events such as earthquakes. Under the dynamic loading conditions induced by earthquakes, bridges may experience abnormal structural responses, potentially leading to severe damage or even failure. This creates substantial challenges for maintaining the safety and integrity of bridge structures, especially when faced with large-scale seismic events. Therefore, the accurate and intelligent automatic identification of earthquake-induced structural responses has become an important research topic in engineering science [1].

On the one hand, effective seismic vibration identification can detect earthquakes in time, so intelligent extraction of earthquake excitation data monitored by sensors is of profound significance for real-time earthquake damage assessment and structural earthquake damage diagnosis [2,3]. On the other hand, effective structural vibration response identification methods are also beneficial to collecting and mining key information in massive and reliable data to meet the data needs of future simulation model revision, neural network training, and even the development of digital twin technology. Identification accuracy is a key factor in earthquake identification [4]. Traditional vibration recognition methods usually require complex algorithms to extract features, which show low performance and low efficiency.

In recent years, thanks to the large-scale construction of bridge health monitoring systems, the limitations of traditional monitoring systems in terms of coverage and quantity have been broken through. The monitoring system has obtained a large number of earthquake excitation and its corresponding structural response data samples. These data provide a valuable basis for establishing structural vibration response identification models. In this context, Cardoni, Elahi, and Cimellaro [5] proposed a refined automated frequency domain decomposition (AFDD) method using the modal assurance criterion (MAC) to improve the accuracy of modal identification, showing that the approach could better extract modal properties from structural data in bridge health monitoring systems. Similarly, Stoura et al. [6] introduced the Dynamic Partitioning Method (DPM) to solve the vehicle-bridge interaction (VBI) problem, highlighting its ability to dynamically model the coupled vehicle–bridge system and improve computational efficiency in large-scale analyses. Additionally, Yang et al. [7] developed a free-vibration detection technique based on iterative variational mode decomposition, demonstrating that the method could effectively separate forced and free vibrations, enabling reliable modal identification and structural state assessment for railway bridges.

For the structural response information mining method based on data-driven and feature extraction ideas, the main advantages are that data acquisition, collection, and processing are convenient, and can truly reflect the structural response characteristics during earthquakes; the disadvantage is that existing structural seismic response samples are limited, which is in-depth learning. Overfitting is prone to occur during model training, so it is necessary to cooperate with signal processing technology to eliminate redundant features in the samples and retain the core vibration characteristics. Additionally, methods like the DPM for VBI provide a dynamic and cost-efficient way to model complex interactions, complementing seismic response analysis by addressing the coupled dynamics of vehicle and bridge systems.

Deep learning algorithms have developed rapidly in recent years. For classification algorithms, both sequence recognition and image recognition methods have shown excellent performance [8]. Among them, long-term and short-term memory neural networks can accurately identify time series data and avoid disappearance and explosive gradient problems [9]. Convolutional neural networks are highly invariant to image translation, scaling, and distortion, and are widely used in image recognition [10,11].

To sum up, this paper proposes an innovative method that combines signal processing technology with deep learning technology. First, signal processing technology is used to effectively extract features from bridge vibration signals; then, these signal features are classified through deep learning neural networks to achieve high-precision identification of structural vibration types. This method is expected to provide a new and efficient technical method for bridge structural health monitoring and earthquake engineering.

## 2. Data Signal Feature

Because seismic signals are complex non-stationary signals and are affected by many factors, it is difficult to identify simply by information such as the amplitude of sensor data. During the running of high-speed railway trains, large vibrations will occur on the rails and piers. This vibration can be used for high-speed railway fault diagnosis and operation safety testing, and can even generate high-speed railway seismic wavefields that can be detected several kilometers away. Therefore, the main difficulty in identifying the seismic response of high-speed railway bridges is to distinguish the source of the vibration signals collected by sensors installed on the bridge structure, especially the vibration response caused by the earthquake from the vibration response generated during the running of the train.

### 2.1. Source of Bridge Earthquake Response Samples

**Figure 1:**
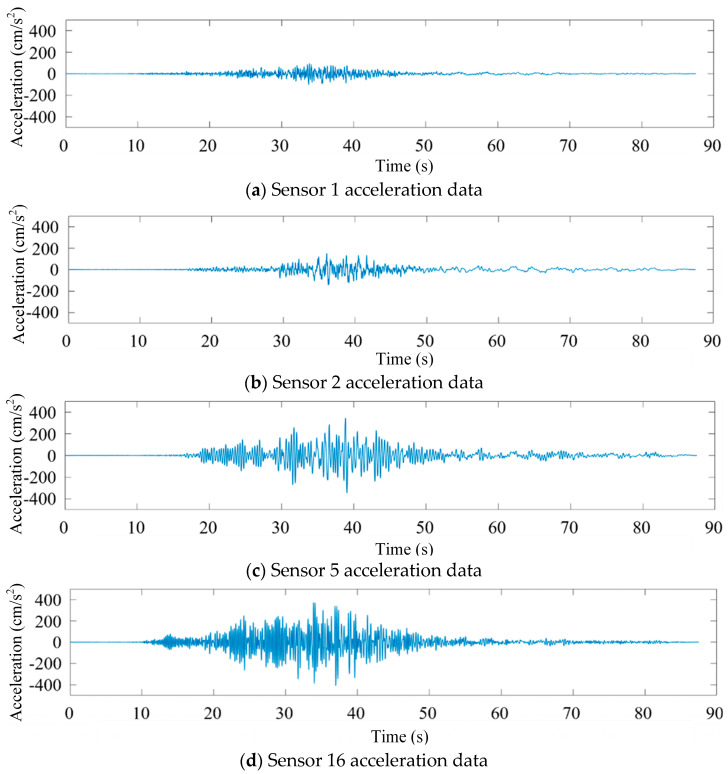
Partial acceleration data map of Meloland overpass bridge.

In order to obtain sufficient vibration data on the seismic response of bridge structures, structural acceleration responses in the CESMD database were used as a sample source [12]. Take the Meloland Flyover Bridge (No. 58-215) collected in the database as an example. Its recorded station number is 01336. For example, the vibration signals collected by the bridge under the earthquake named Calexico (ID in the database: ci14607652) on 4 April 2010 are taken as an example. The magnitude of the earthquake is 7.2 ML, the ground PGA is 0.213, and the structure PGA is 0.474. Some sensor data are shown in Figure 1. Among them, sensors No. 2 and No. 1 are horizontal and vertical sensors installed on the foundation of the bridge pier, respectively, and sensors 5 and 16 are, respectively, horizontal and vertical sensors installed in the middle of the main beam span.

### 2.2. Sample Sources of Vehicle-Induced Vibration Response of Bridges

For the vibration response of railway bridges during train running, vehicle-induced vibration acceleration data collected by a railway bridge health monitoring system is used as a sample. Short-term energy is one of the most basic short-term parameters of a signal. It characterizes the energy of a frame of signal and is an important time-domain feature of the signal. Since there is a significant difference in short-term energy between effective signals and noise signals, short-term energy analysis can be used to segment and extract non-stationary data, and data cleaning can be carried out at the same time.

**Figure 2:**
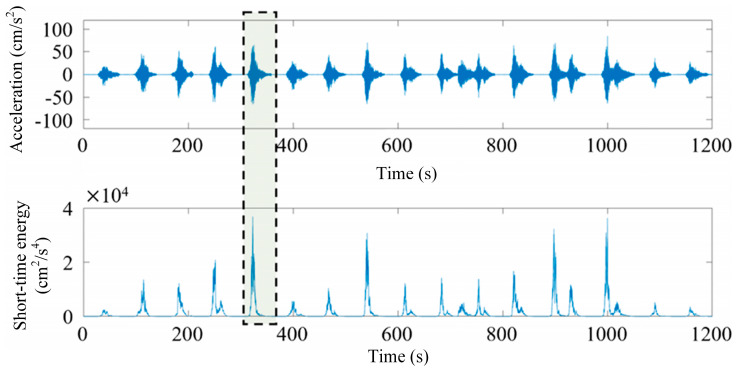
Raw signal and the corresponding short-term energy analysis results.

Figure 2 shows a 1200 s raw signal collected on 7 June 2017 by the sensor JSD-15-06 installed in the center of the main beam in the mid-span section and the corresponding short-term energy analysis results.

To facilitate the subsequent use of deep learning methods for identifying the seismic response of bridges, for each non-stationary segment, the 30 s data segment with the maximum short-term energy is extracted as a sample. This process helps exclude time-dependent features from the neural network model during training. Similarly, for each seismic response data set of the bridge, the 30 s data segment with the maximum short-term energy is also selected as a sample.

**Figure 3:**
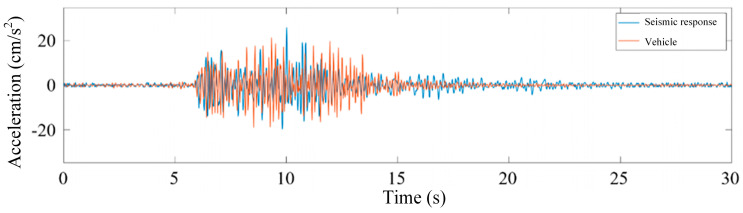
Seismic response vehicle-induced vibration response comparison graph (short-term energy alignment).

The extraction results of bridge seismic response and vehicle-induced vibration response samples are shown in Figure 3. After aligning the short-term energy peaks of the samples, the waveforms of bridge seismic response samples with similar amplitudes and vehicle-induced vibration response samples are very close.

## 3. Feature Extraction Method of Earthquake Response Signal

Due to the low frequency of earthquakes with higher magnitudes and the sparse structure monitoring networks, the amount of obvious structural seismic response data that can be collected and used in the past is very limited. In order to break through the limit of the number of response samples, we can use methods such as feature extraction to mine the most valuable information in limited samples to reduce the sample size required for deep learning technology. In this section, signal processing technology will be used to effectively extract structural vibration characteristics to achieve high-precision identification of structural vibration types.

### 3.1. Short-Time Fourier Transform (STFT)

For the traditional Fast Fourier [13] Transform (FFT) method, it is easy to find that spectrum analysis converts a time-domain signal into a frequency-domain signal, showing the frequency components of the vibration signal while concealing the time-domain characteristics of the signal, which easily leads to large differences in the time domain. Signal spectrum maps are similar.

In order to solve the problem that the fast Fourier transform cannot retain both time domain and frequency domain information, the entire time domain process can be decomposed into several approximately smooth small processes by introducing a window function, and the short-time Fourier transform (STFT) is used to extract the time–frequency domain characteristics of the vibration signal. Short-term Fourier change can be defined by Equation (1), as follows:

(1) $$X(\tau ,\omega )=\int_{-\infty }^{\infty }x(t)\varpi (t-\tau )e^{-j\omega t}dt$$

where τ is the location where the window function is added, ω is the frequency, x(t) is the input signal, and ϖ(t) is the window function.

**Figure 4:**
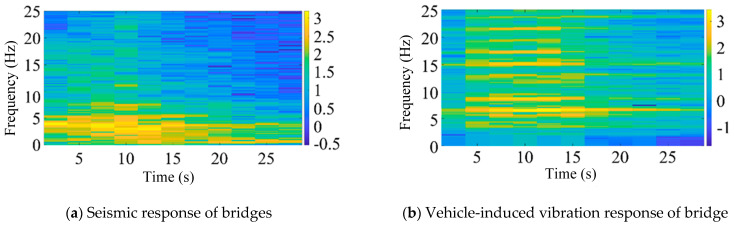
Short-time Fourier transform feature extraction results (logarithmic plot).

According to the above short-time Fourier transform method, a window width of 256 was selected for spectrum analysis, and feature extraction was carried out on the bridge earthquake response samples and the bridge vehicle-induced vibration response samples, respectively. The feature extraction results are shown in Figure 4 (logarithmic plot).

### 3.2. Continuous Wavelet Transform (CWT)

In order to achieve dynamic resolution, the continuous wavelet transform introduces a wavelet mother function based on the Fourier transform, and changes the infinitely long trigonometric function basis used in the Fourier transform to a finite-length attenuated wavelet basis. This enables spectrum analysis with dynamic resolution. Take the morlet wavelet basis function as an example. It is composed of a complex trigonometric function multiplied by an exponential decay function. Its expression is as shown in Equation (2):

(2) $$\psi (t)=\exp(j\omega_{0}t)\exp(-\frac{t^{2}}{2})$$

where $\omega_{0}$ represents the center frequency and t represents the time variable. Then, the wavelet transform can be expressed as Equation (3), as follows:

(3) $$WT(a,\tau )=\frac{1}{\sqrt{a}}\int_{-\infty }^{\infty }x(t)*\psi (\frac{t-\tau }{a})dt$$

where a represents the size parameter, τ represents the amount of translation, and t represents the time variable.

**Figure 5:**
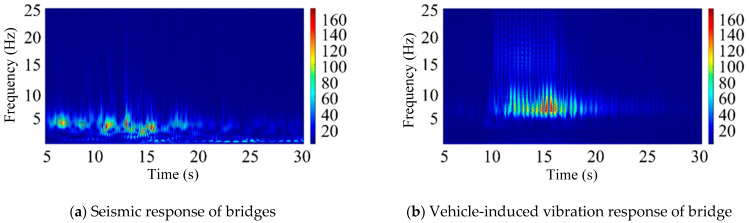
CWT feature extraction results for vibration signal analysis.

According to the above continuous wavelet transform method, feature extraction is carried out on the bridge earthquake response samples and the bridge vehicle-induced vibration response samples, respectively. The feature extraction results are shown in Figure 5.

Figure 5 presents the vibration signal spectrum after CWT. In the image, the red regions correspond to higher vibration energy, while the blue regions indicate lower vibration energy. Compared to the STFT, the CWT offers more refined division in both the time and frequency domains. Specifically, it provides better frequency resolution in the low-frequency region and better time resolution in the high-frequency region. This makes it more suitable for identifying structural responses under seismic excitation, particularly those at lower vibration frequencies. From the figure, it can be observed that, under seismic excitation, the vibration response spectrum of high-speed railway bridges exhibits a concentration of red regions around frequencies of 5 Hz and below. In contrast, the vibration response spectrum under train excitation shows red regions concentrated in frequency bands above 5 Hz. When the seismic and train-induced responses are combined, red-highlighted areas appear in both the sub 5 Hz and above 5 Hz frequency bands.

### 3.3. Mel Frequency Cepstral Coefficient (MFCC)

Mel frequency cepstral coefficients are proposed based on human hearing and are the most commonly used feature extraction method in the field of speech recognition. Since the Mel frequency cepstral coefficients correspond non-linearly to frequency, rather than simply using physical knowledge to express the sound wave characteristics, its non-linear transformation characteristics can effectively remove noise [14]. Considering that compared with commonly used sensors with sampling frequencies of 50 Hz, 100 Hz, 200 Hz, or even higher, the main frequency of seismic waves and the natural vibration frequency of bridge structures are generally lower, the Mel frequency cepstrum coefficient method can be used to go from low to high frequency. In this band, a set of band-pass filters are set from dense to sparse according to the critical bandwidth to filter the input signal. The signal energy output by each bandpass filter is used as the basic feature of the signal, and after further processing, this feature can be used as the input feature for vibration recognition. Because this feature does not depend on the nature of the signal, does not make any assumptions or restrictions on the input signal, and fully considers the vibration characteristics of the structure, it has better robustness and still has good recognition performance when the signal-to-noise ratio is reduced.

The conversion formula between frequency and Mel frequency is Equation (4), as follows:

(4) $$\left\{\begin{array}{l} M(f)=1125\ln(1+f/700)\\ M^{-1}(m)=700(\exp(m/1125)-1) \end{array}\right.$$

where f represents the frequency and m represents the Mel frequency value.

After filtering through a Mel filter bank, the Mel spectrum is obtained, and cepstrum analysis is performed on the Mel spectrum (taking the logarithm and then performing inverse transformation, which is generally achieved by discrete cosine transform). Take the logarithm of the vector and perform the discrete cosine transform. The discrete cosine transform is as shown in Equation (5).

(5) $$c_{i}=a_{i}\sqrt{\frac{2}{N}}\sum_{j=1}^{N}m_{j}\cos(\frac{\pi i}{N}(j-0.5))$$

where the value of a is as shown in Equation (6), as follows:

(6) $$a_{i}=\left\{\begin{array}{l} \sqrt{\frac{1}{N}},n=0\\ \sqrt{\frac{2}{N}},n=1,2,\ldots ,N-1 \end{array}\right.$$

where *N* represents the order of change and is usually equal to the length of the input vector.

The 2nd to 13th coefficients after discrete cosine transform are often taken as MFCC coefficients. Since the signal is continuous in the time domain, the feature information extracted in frames only reflects the characteristics of the signal in this frame. In order to make the features more reflect the continuity of the time domain, the dimension of the front and back frame information can be increased in the feature dimension through a first-order difference or second-order difference to complete dynamic feature extraction.

**Figure 6:**
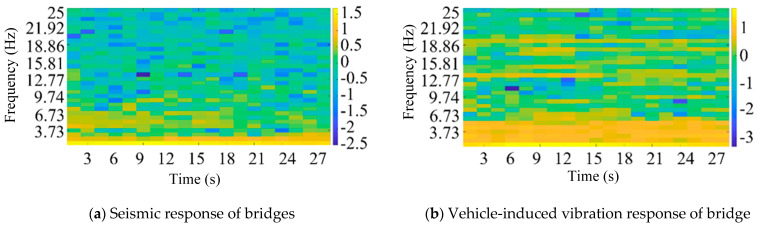
Feature extraction results of Mel frequency cepstrum coefficient (logarithmic plot).

According to the above Mel frequency cepstrum coefficient method, feature extraction is carried out on the bridge earthquake response samples and the bridge vehicle-induced vibration response samples, respectively. The feature extraction results are shown in Figure 6 (logarithmic plot).

Figure 6 presents the logarithmic power spectrum of the vibration signal after Mel frequency wrapping. The brighter regions in the image correspond to higher vibration energy, which is concentrated in the low-frequency bands, facilitating signal identification. By applying the discrete cosine transform (DCT) to the logarithmic spectrum, a multi-frequency envelope of the MFCC matrix is obtained. The size of the matrix is primarily determined by factors such as the frame length, frame shift, signal length, and the number of bands in the bandpass Mel scale filter, and it can be adjusted according to the specific requirements of the analysis.

## 4. Seismic Response Identification Method Based on Deep Learning

The dataset used in this section for the deep learning network has a total of 3000 samples, including 600 samples of bridge seismic response and 2400 samples of bridge vehicle-induced vibration response. The ratio of the seismic response to vehicle-induced vibration response samples is 1:4 (since the main goal of response identification is the bridge seismic response, the seismic response is merged into the vehicle-induced vibration response samples and classified into the bridge seismic response samples). The ratio of the training set, verification set, and test set is 7:2:1.

### 4.1. Identification Method Based on Sequence Classification

Long short-term memory (LSTM) is an improved recurrent neural network. By introducing gate functions into the cell structure to solve long-term dependence, it can make more full use of longer time series data. At the same time, the long-term memory neural network can also meet the requirements of time dependence between explanatory variables and highly nonlinear expression. Unlike recurrent neural networks, the cell structure and internal data flow in the hidden layers of long-short-term memory neural networks are more complex.

An LSTM cell contains three main gates: the forget gate, the input gate, and the output gate. The forget gate decides how much of the previous memory should be discarded, the input gate determines how much new information should be added, and the output gate controls the amount of memory to be output. At each time step, the LSTM updates the cell state and hidden state through these gate mechanisms, effectively capturing both short- and long-term dependencies. Specifically, the forget and input gates regulate the flow of information using weighted inputs and the previous state, updating the cell state, while the output gate generates the new hidden state based on the current cell state. This architecture addresses the long-term dependency issue in traditional RNNs, making it more effective for handling time-series data.

After inputting the time series into the LSTM network, for a long-short-term memory neural network cell, the input data include the current moment information after processing deviations and weights, the information transmitted by the long-short-term memory neural network cell at the previous moment, and the flowing long-term memory in the cell. These weights, deviations, and activation functions are called the “forgetting gate”, “input gate”, and “output gate”. The forgetting gate determines what information is lost in the cell, the input gate determines what information is updated in the cell, and the output gate determines what information is output from the cell.

**Figure 7:**
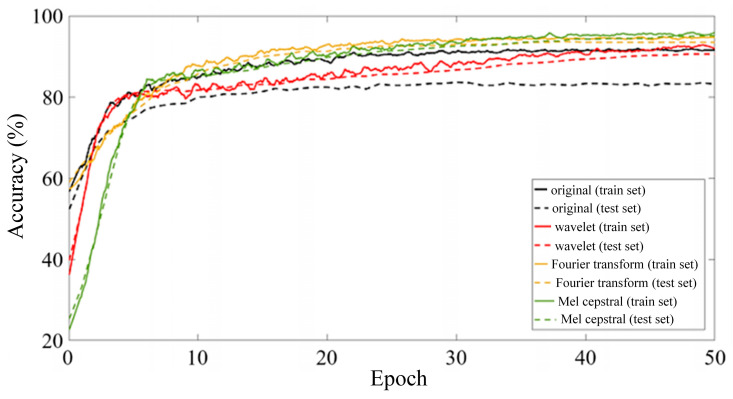
Iteration process diagram of long-term and short-term memory neural network.

Since the capacity of a single LSTM unit is limited when building a model, the LSTM unit must be organized into a specific network architecture when processing actual data. The LSTM network architecture has a significant impact on the performance and accuracy of the network. In addition, the selection of hyperparameters also has a significant impact on the training effect of LSTM networks, especially since some hyperparameters, such as the batch size and learning rate, are decisive for the final convergence result of the network [15]. Considering the data size and equipment computing power, and controlling variables to compare the feature extraction effects of various signal processing algorithms, this paper sets the Batch size to 50, uses the Adam optimizer to carry out backward propagation, and performs a total of 50 rounds of iteration. The initial learning rate is set to 0.0001 to ensure the convergence of the LSTM network. The changes in the recognition accuracy of the feature matrices obtained by each signal processing method during 50 iterations of the LSTM network are shown in Figure 7.

**Figure 8:**
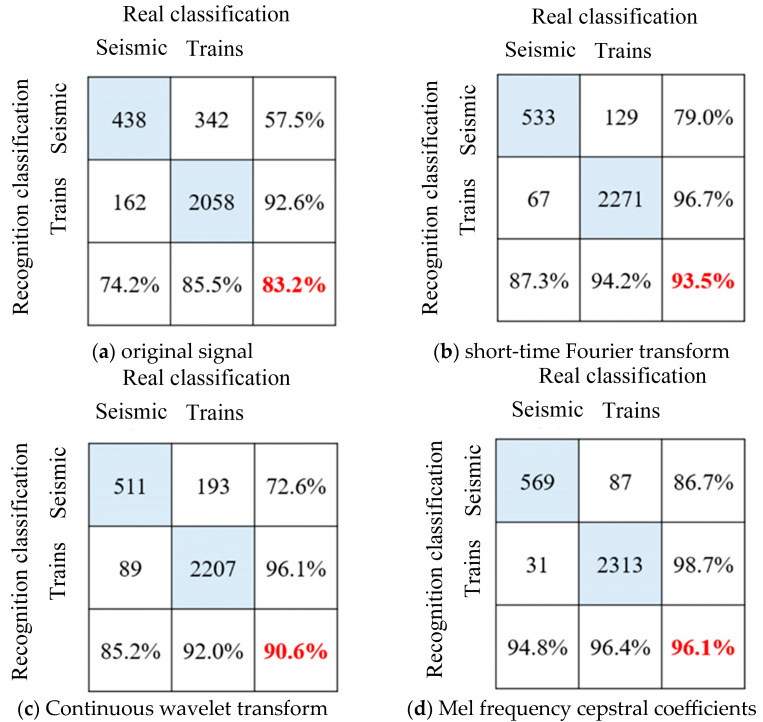
Long-short-term memory neural network sequence recognition results.

It can be seen from Figure 7 that after 50 rounds of iteration, the identification accuracy of each LSTM model tends to be stable and the model training converges. The original vibration signal showed obvious overfitting during the training process, with an accuracy rate of 91.6% for the training set and 83.2% for the verification set. After signal processing, the overfitting phenomenon is significantly improved, and the test set accuracy is significantly improved. The model test set accuracy of the three feature extraction methods is shown in the confusion matrix in Figure 8. Among them, the Mel cepstrum coefficient method has the highest recognition accuracy and the continuous wavelet transform method has the lowest recognition accuracy.

### 4.2. Recognition Method Based on Image Recognition

With the continuous development of Convolutional Neural Networks (abbreviated as CNNs), in order to obtain deep-level features, the number of convolution layers is increasing. At first, the LeNet network had only five layers, and then AlexNet had eight layers. Later, the VggNet network contained 19 layers, and GoogleNet already had 22 layers. However, it is not always feasible to enhance the learning ability of the network by increasing the number of network layers, because after the number of network layers reaches a certain depth and then increases the number of network layers, the network will have the problem of the disappearance of random gradients, which will also lead to a decrease in the accuracy of the network. In order to solve this problem, the traditional method uses data initialization and regularization methods, which solves the problem of gradient disappearance, but the problem of network accuracy has not been improved.

The emergence of residual networks can solve the gradient problem, and the increase in the number of network layers also makes it express better characteristics, and the corresponding detection or classification performance is stronger. In addition, a 1 × 1 convolution is used in the residual networks. This can reduce the number of parameters and reduce the amount of calculation to a certain extent. The key to the ResNet network lies in the residual unit in its structure. The residual network unit contains cross-layer connections. The direct mapping part in the figure can directly pass the input across layers, perform the same mapping, and then add the residual results to the convolution operation. The residual part usually contains a five-layer structure, namely two Weight Layers (usually using the convolution layer Conv), two Regularization Layers (Batch Normalization), and an activation function layer (the Resnet50 network uses the Relu function as the activation function).

Assume that the input image is $x_{l}$ and the output is $H(x_{l})$. After convolution, the output is the non-linear function $F(x_{l},W_{l})$ of the residual part. The final output is $H(x_{l})=F(x_{l},W_{l})+h(x_{l})$, which allows for further non-linear transformations. The residual refers to the “difference”, which is $F(x_{l},W_{l})$. Therefore, the network essentially transforms into optimizing the residual function $F(x_{l},W_{l})=H(x_{l})-h(x_{l})$. This residual function is clearly easier to optimize compared to directly optimizing $F(x_{l},W_{l})=H(x_{l})$.

**Figure 9:**
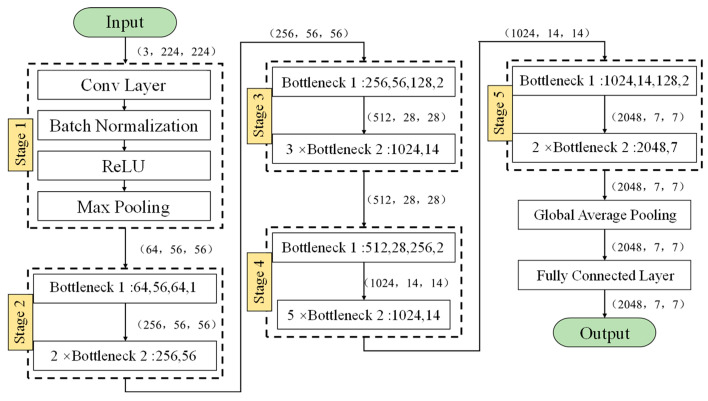
Overall architecture of the ResNet network.

The Resnet50 network contains 49 convolutional layers and a fully connected layer. The Resnet50 network structure can be divided into seven parts, as shown in Figure 9. The first part does not contain residual blocks, and mainly performs convolution, regularization, activation functions, and maximum pooling calculations on the input. The second, third, fourth, and fifth parts of the structure all contain residual blocks (i.e., “bottlenecks”). In the Resnet50 network structure, each residual block has three layers of convolution. Add the first part of a convolution layer and the last fully connected layer, and there are 50 convolution layers in total, which is also the name Resnet50. The input of the network is 3 × 224 × 224. After the first five parts of the convolution calculation, the output is 2048 × 7 × 7. The pooling layer will convert it into a feature vector, and finally the classifier will calculate this feature vector and output the category probability.

In order to further improve the recognition accuracy, when using the Resnet50 network for image recognition, the feature matrix map needs to be preprocessed first: First, remove information such as the map name, coordinates, and legend to reduce invalid features that cannot be used for classification and recognition. Second, the unified image size is 224 × 224 × 3 pixels, which gives full play to the easy-to-segmentation of low-resolution images. At the same time, it can reduce the computing power required by neural networks and improve computing efficiency.

**Figure 10:**
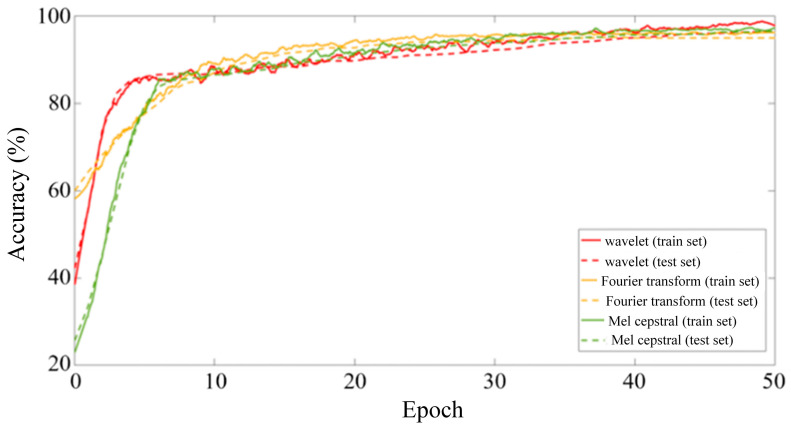
ResNet50 neural network training and iteration process for vibration signal classification.

Considering the data size and equipment computing power, and controlling variables to compare the feature extraction effects of various signal processing algorithms, same as the LSTM neural network, this paper sets the Batch size to 50, uses the Adam optimizer to carry out backward propagation, and carries out a total of 50 rounds of iteration. The initial learning rate is set to 0.0001 to ensure that the Resnet50 network converges. The changes in the recognition accuracy of the feature matrices obtained by each signal processing method during 50 iterations of the Resnet50 network are shown in Figure 10.

**Figure 11:**
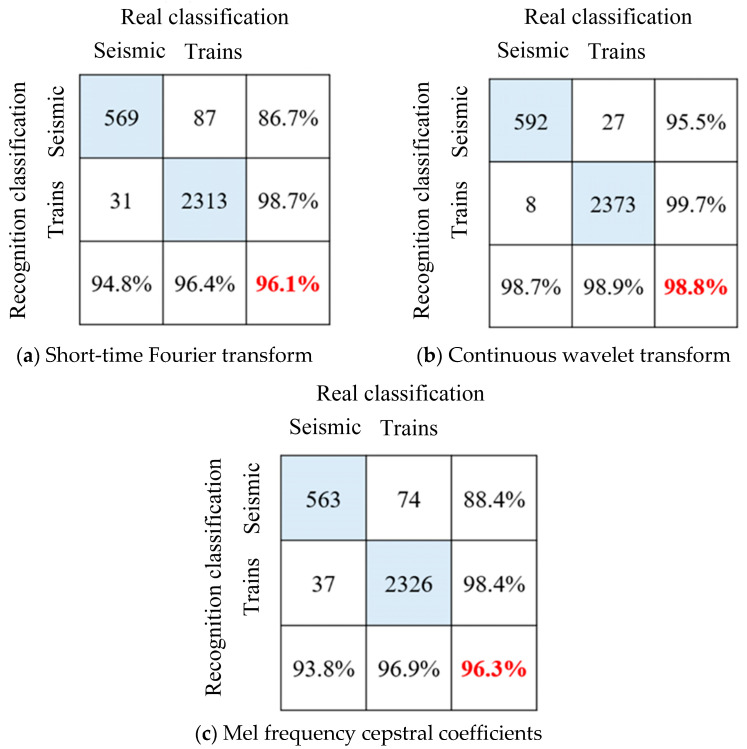
ResNet50 neural network image recognition results for vibration signal classification.

It can be seen from Figure 10 that after 50 rounds of training, after using the feature matrix map as a sample and reducing the image resolution, the overfitting phenomenon is further improved, and the accuracy rate is increased to 98.8%. The model test set accuracy of the three feature extraction methods is shown in the confusion matrix in Figure 11. Compared with time series classification methods, the accuracy is improved, especially the continuous wavelet transform method. It is proved that drawing time–frequency maps and reducing pixels does reduce the number of features, and model training under limited samples is more effective.

### 4.3. Comparison of Earthquake Response Identification Accuracy

Table 1 shows the comparison results of the earthquake response identification accuracy and efficiency of various signal processing methods and deep learning networks. Specifically, the time–frequency graph obtained by drawing the time–frequency feature matrix into a time–frequency graph has the same specification by the last three feature processing methods. The original feature matrix of continuous wavelet transform is large, and the feature reduction is the most obvious after drawing. Therefore, the accuracy of the continuous wavelet transform is improved most after using the image recognition method. However, the feature matrix obtained by the Mel frequency cepstrum coefficient method is small, so the feature reduction effect is not obvious after drawing time–frequency maps.

Comparing the sample test time and sample memory occupation under various methods, we can draw the following conclusion: in the field of real-time monitoring, compared with the 30 s length of a sample, the difference in the test time between each method is not obvious; in order to improve the accuracy of algorithm recognition, the image recognition method using the Resnet50 neural network + continuous wavelet transform, which occupies a slightly larger sample memory, is acceptable. However, in large-scale data identification and acquisition applications, the sequence classification method of LSTM neural network + Mel frequency cepstral coefficients, which loses some accuracy in exchange for a faster test time and smaller memory occupation, is more cost-effective.

## 5. Conclusions

In this paper, the CESMD database is used as monitoring data sources, and three signal feature extraction methods: the continuous wavelet transform, short-time Fourier transform, and Mel cepstral coefficient are used. Based on the LSTM neural network and Resnet50 neural network, a method for identifying earthquake vibration signals is established. The specific conclusions are as follows:

(1) Under the limit of the number of samples, compared with the LSTM neural network model established with the original vibration signal, the earthquake vibration recognition rate of the LSTM neural network model established based on the signal processing method is significantly improved, which proves that the signal processing method can effectively extract features and optimize model overfitting;
(2) Compared with the temporal classification under the LSTM neural network model, the image recognition and classification method based on the Resnet50 neural network model has better feature extraction capabilities under small samples, mainly due to the significant reduction in the number of sample features;
(3) In the field of real-time monitoring, the image recognition method based on the Resnet50 neural network + continuous wavelet transform can achieve higher recognition accuracy; in large-scale data recognition and acquisition applications, the sequence classification method based on LSTM neural network + Mel cepstral coefficients has higher cost performance.

However, during the research process of this paper, in order to control variables and compare the identification accuracy of multiple methods, each neural network architecture is common, but the architecture and parameters of the neural network model are not specifically optimized for specific signal processing methods. In subsequent research, further architecture and hyperparameter optimization can be carried out for the neural network model to further improve the recognition accuracy.

## Figures and Tables

**Table 1:** Comparison of identification accuracy and efficiency of various methods.

Neural Network	Signal Processing Method	Sample Specifications	Test Time for 1000 Samples (s)	1000 Samples Consume Memory (MB)	Test Set Accuracy
NLSTM	Original data	1×1500 double	0.92	10.94	83.20%
	Continuous wavelet transform	150×1500 double	5.25	1625.00	90.60%
	Short-time Fourier transform	129×11 complex double	0.92	21.00	93.50%
	Mel cepstral coefficient	22×32 double	0.90	5.24	96.10%
Resnet50	Continuous wavelet transform	224×224×3 pixel	5.80	7.35	98.80%
	Short-time Fourier transform	224×224×3 pixel	5.80	7.38	96.70%
	Mel cepstral coefficient	224×224×3 pixel	5.82	7.37	96.30%

## Data Availability

The raw data supporting the conclusions of this article will be made available by the authors on request.
